# Arthrite à pneumocoque chez un adulte immunocompétent

**DOI:** 10.11604/pamj.2015.21.139.6421

**Published:** 2015-06-22

**Authors:** Hicham Chemsi, Maryama Chadli, Yassine Sekhsokh

**Affiliations:** 1 Laboratoire de Recherche et de Biosécurité, HMIMV, Faculté de Médecine et de Pharmacie de Rabat Université Mohammed V-Souissi, Maroc; 2 Service de Bactériologie HMIMV, Faculté de Médecine et de Pharmacie de Rabat, Université Mohammed V-Souissi, Maroc

**Keywords:** Arthrite rhumatoïde, immunocompétent, arthrite à Pneumocoque, rheumatoid arthritis, immunocompetent, Pneumococcal arthritis

## Abstract

Les infections à pneumocoques sont avant tout respiratoires, ORL et méningées. Les infections ostéoarticulaires à pneumocoque ont la particularité de survenir dans moins de 20% des cas chez l'adulte sain. Habituellement, un ou plusieurs facteurs favorisants sont retrouvés. Toutefois, nous rapportons lors de cette observation le cas d'une arthrite à *Streptococcus pneumoniae* chez un adulte immunocompétent sans facteurs prédisposant. Patient âgé de 66 ans, diabétique de type II, a été hospitalisé pour une décompensation acido-cétosique et une monoarthrite du genou droit. Ce patient était fébrile (39°C) et présentait un genou droit inflammatoire en flexion avec rougeur et chaleur locale et un choc rotulien. Une ponction articulaire avec d'autres examens ont été réalisés pour confirmation d'une arthrite septique à pneumocoque. Le résultat de la ponction articulaire réalisée a montré un liquide jaune citron trouble avec 480 000 leucocytes/mm^3^ à prédominance polynucléaires neutrophiles. L'examen direct a montré des coccis à Gram positif en diplocoque, la culture a permis d'isoler un *Streptococcus pneumoniae* sensible à la pénicilline G. L’évolution clinique et biologique de l'arthrite du genou était favorable. Un déficit immunitaire, un asplénisme anatomique ou fonctionnel peuvent être en cause. L'alcoolisme est un facteur favorisant mais le mécanisme n'est pas clairement élucidé. La présence de matériel prothétique, peut favoriser une localisation septique. Ces facteurs de risque doivent être systématiquement recherchés, notamment en cas d'infection grave ou récidivante, une antibioprophylaxie ou une vaccination pouvant être proposées chez les sujets à haut risque.

## Introduction

Le *Streptococcus pneumoniae* est un agent pathogène habituel chez l'homme. Il provoque des infections de l'oreille moyenne, des voies aériennes supérieures et des poumons par extension locale directe à partir d'une colonisation nasopharyngée. La dissémination hématogène à d'autres organes, appelée infection systémique à pneumocoque (ISP), est moins habituelle mais était la principale cause de morbidité et de mortalité avant l’ère des antibiotiques [1]. L'arrivée des antibiotiques dans les années 1930 a provoqué une diminution rapide des infections à pneumocoque de localisations inhabituelles et de la mortalité liée aux ISP. Cependant, il a été observé lors des dernières années une augmentation des ISP, probablement en rapport avec l’émergence de souches de *S. pneumoniae* résistantes à la pénicilline, en particulier chez les enfants et les patients infectés par le virus de l'immunodéficience humaine (VIH) [2]. Ainsi les arthrites à pneumocoques ne représentent que 5 à 6% des infections à pneumocoques [3]. Nous rapportons le cas d'une arthrite à pneumocoques chez un adulte immunocompétent.

## Patient et observation

Patient âgé de 66 ans, diabétique de type II, sous antidiabétiques oraux type glymépiride depuis 3ans, a été hospitalisé pour une décompensation acido-cétosique et une monoarthrite du genou droit. Ce patient avait comme antécédents notoires un tabagisme chronique (80 paquets/an) sevré depuis 13 ans et une arthrite fugace du genou droit. A l'admission le patient était fébrile (39°C), il présentait un genou droit inflammatoire en flexion avec rougeur et chaleur locale et un choc rotulien. . La ponction articulaire effectuée a montré un liquide jaune citron trouble avec 480 000 leucocytes/mm^3^ à prédominance de polynucléaires neutrophiles. L'examen direct a montré des cocci à Gram positif en diplocoque, la culture a permis d'isoler un *S. pneumoniae* sensible à la pénicilline G. D'autres examens biologiques ont été réalisés à savoir l'examen cytobactériologique urinaire qui a révélé l'absence d'infection urinaire, la ponction lombaire a écarté le risque de méningite, l'hémoculture qui était stérile et l'examen cytobactériologique des crachats a révélé la présence d'un germe contaminant *Candida tropicalis*. Les valeurs biologiques étaient les suivantes: Leucocytes 249 000 /µl, Plaquettes 336 000 /µl, Hg 12,7 g/µl, PCR 490 mg/µl, Vs 91 mm/h, Créatinémie 11 mg/µl, ASAT 41 IU /L, ALAT 20 IU /L, LDH 234 /mm^3^. L’échographie Doppler a mis en évidence les séquelles d'une thrombose veineuse profonde associée. Etant donné la localisation atypique du germe, un myélome ainsi qu'une néoplasie profonde ont été recherchés et écartés grâce à une électrophorèse des protéines plasmatiques, par contre une radio pulmonaire standard a révélé deux foyers pulmonaires infectieux. La décompensation acido-cétosique a été prise en charge et l’évolution clinique et biologique de l'arthrite du genou était favorable sous Gentamycine (2g/24h) pendant 6 jours et Céftriaxone (2g/24h) pendant 5 semaines.

## Discussion

Comme il a été souligné, le *S. pneumoniae* est principalement incriminé dans les infections de type ORL, broncho-pulmonaires ou méningées, mais il peut également être responsable d'infections ayant d'autres localisations comme les arthrites. Ces infections sont fréquemment bactériémiques avec des hémocultures positives dans plus de 70% des cas. L'atteinte est pluriarticulaire dans 30% des cas [4]. Leur taux de mortalité est comparable à celui des autres agents pathogènes, variant de 20 à 30% selon les études [4, 5]. L’évolution dépend de la sévérité de l'atteinte, de sa chronicité, et du terrain. Les infections ostéo-articulaires à pneumocoque ont la particularité de survenir dans moins de 20% des cas chez des adultes sains. Habituellement, un ou plusieurs facteurs favorisants sont retrouvés [6]. Un déficit immunitaire primitif ou iatrogène (corticothérapie, immunosuppresseurs), une affection modifiant l'immunité (hémopathie, sida) ou un asplénisme anatomique ou fonctionnel (splénectomie, drépanocytose) peuvent être en cause. L'alcoolisme est un facteur favorisant classiquement évoqué mais le mécanisme n'est pas clairement élucidé. Une atteinte articulaire préalable, d'origine inflammatoire (polyarthrite rhumatoïde) ou la présence de matériel prothétique, peut favoriser une localisation septique, ces facteurs de risque doivent être systématiquement recherchés, notamment en cas d'infection grave ou récidivante, une antibioprophylaxie ou une vaccination pouvant être proposée chez les sujets à haut risque [7]. Toutefois, notre observation montre que des sujets apparemment sans facteur prédisposant peuvent développer des infections articulaires sévères à pneumocoque. D'autres facteurs de risque restent probablement à découvrir. Des anomalies de l'immunité innée, en particulier de la voie des TLR (« Toll-likereceptor »), ont ainsi été récemment évoquées. Currie et al. [8] ont décrit l'observation originale d'un enfant de trois ans souffrant d'infections récidivantes à pneumocoque chez qui l’étude des monocytes a permis de détecter un défaut de production de cytokines dans la voie des TLR. Le cas que nous avons décrit montre que le patient est infecté par un *S. pneumoniae* sensible aux pénicillines. Cependant, l’émergence des souches de pneumocoque de sensibilité diminuée à la pénicilline (PSPD), pose le problème du traitement des infections ostéoarticulaires impliquant ce germe. L'Amoxicilline peut se révéler en théorie insuffisante même si aucune corrélation n'a été démontrée à ce jour entre résistance à la pénicilline in vitro et échec clinique et mortalité associés à ce traitement [9].

Les recommandations thérapeutiques des pneumopathies et des méningites bactériennes ont en conséquence été modifiées privilégiant l'utilisation des céphalosporines de troisième génération (C3G) en première intention, voire des glycopeptides, tant qu'un PSDP n'est pas écarté [10]. La gravité potentielle des infections ostéoarticulaires à pneumocoque conduit certains auteurs à envisager une attitude identique [4]. Les nouvelles fluoroquinolones à spectre élargi sur le pneumocoque (Levofloxacine, Moxifloxacine) utilisées en alternative à la pénicilline dans le traitement des infections pulmonaires [11], pourraient également être proposées. Elles possèdent en effet une bonne efficacité sur *S. pneumoniae* indépendamment de sa sensibilité aux bêtalactamines et une excellente diffusion osseuse et articulaire. L'antibiothérapie pourrait également être guidée par l’étude des facteurs de risque d'infection à PSDP: un âge supérieur à 65 ans, une prise de bêtalactamines dans les trois mois précédents, un éthylisme, une immunodépression, des co-morbidités multiples, une exposition à un enfant vivant en collectivité.

## Conclusion

*S. pneumoniae* doit être évoqué devant toute infection ostéo-articulaire, même en l'absence des facteurs favorisants classiquement associés. Ceux-ci doivent être recherchés systématiquement, en particulier en cas d'infections sévères ou récidivantes. Par ailleurs, une révision des recommandations thérapeutiques concernant les infections ostéoarticulaires peut être suggérée devant l'augmentation de la prévalence des PSDP et le développement récent de nouvelles molécules d'antibiotiques. Une plus grande place pourrait ainsi être laissée aux C3G et aux nouvelles fluoroquinolones, en particulier l'existence des signes de gravité et des facteurs de risque d'infection pneumococcique.
